# Bringing context to balance: development of a reactive balance test within the injury prevention and return to sport domain

**DOI:** 10.1186/s40945-019-0057-4

**Published:** 2019-04-16

**Authors:** Jo Verschueren, Bruno Tassignon, Bert Pluym, Jeroen Van Cutsem, Evert Verhagen, Romain Meeusen

**Affiliations:** 1 Faculty of Physical Education and Physiotherapy, Human Physiology Research Group, Vrije Universiteit Brussel, Brussels, Belgium; 2 Amsterdam Collaboration on Health and Safety in Sports, Department of Public and Occupational Health, Amsterdam Movement Sciences, Amsterdam UMC, Vrije Universiteit Amsterdam, Van der Boechorststraat 7, NL-1081 BT Amsterdam, The Netherlands

**Keywords:** Balance, Visuomotor reaction time, Adaptability, Stability, Injury prevention, Return to sport

## Abstract

**Background:**

Balance tests are commonly used in clinical practice with applicability in injury prevention and return to sport decisions. While most sports injuries occur in a changing environment where reacting to a non-planned stimulus is of great importance, these balance tests only evaluate pre-planned movements without taking these dynamics environmental aspects into account. Therefore, the goal of this paper was to develop a clinician-friendly test that respects these contextual interactions and to describe the test protocol of an adapted Y-balance test that includes environmental perception and decision-making.

**Methods:**

Within the theoretical construct of balance and adaptability, balance errors were selected as outcome measures for balance ability and, visuomotor reaction time and accuracy are selected as outcome measures for adaptability. A reactive balance task was developed and described using the Y-balance test for the balance component, while the FitLight training system^TM^ was chosen for the environmental perception and decision-making component of the test.

**Results:**

This paper describes the test protocol of a reactive balance test as an adapted Y-balance test. The LED-lights of the FitLight training systemTM are placed at 80% of the maximal reach distance for each axis along the Y-Balance test kitTM. To induce cognitive load within the visuomotor task, colours were fixed to a corresponding axis, and both the order of the visual stimuli as the interstimulus time were randomised to integrate environmental perception and decision-making.

**Conclusion:**

The reactive balance test is a functional test that allows clinicians to score balance ability and athlete adaptability easily.

## Introduction

Recently, several systematic reviews and clinical commentaries emerged regarding the clinimetric value of clinician-friendly lower extremity functional performance tests, and their associations with injury [1–4]. Although balance is an important part of an athlete’s functional ability [5–7], these reviews showed that balance tests are currently underrepresented, accounting for only 1 functional balance test within the functional testing repertoire of 14 tests. Nevertheless, balance tests are commonly used in the assessment of ankle and knee injury prevention and return to sport decisions in clinical practice [8–14]. Glasgow et al. (2013) illustrated that reacting to a non-planned stimulus is of great importance in sports. They stated that the key driver for effective sporting performance and injury prevention is the athlete’s ability to adapt his or her responses under a comprehensive variety of conditions [15]. This makes the applicability of the outcomes of pre-planned balance tests to open skilled sports (e.g. tennis, football) low, given that static tests neglect the importance of balance in its inherent relation with being able to react to a changing environment.

The adequate and immediate motor reaction to a changing visual stimulus can be seen as a hallmark of an athlete’s adaptability. In this perspective, balance and visuomotor reaction time are two very important and strongly related components of sports performance, and are also correlated to (recurrent) injury risk in a sports context [8, 16–18]. Although reaction time and balance can be measured separately in functional tests [19, 20], a functional test that combines balance and visuomotor reaction time coupled to environmental perception is currently lacking within the clinician-friendly testing repertoire. Since the interaction of balance and visuomotor reaction time may be a key feature in attaining successful sports performance and injury prevention, it was expedient to merge the constructs ‘balance’, ‘environmental perception’ and ‘visuomotor reaction time’ in a clinician-friendly functional test.

Therefore, the goal of this paper was to describe the development of a test protocol for a reactive balance test incorporating environmental perception, decision-making and visuomotor reaction time.

## Methods

The methodology of Kazman et al. (2016) [21] was used to systematically describe the development of the reactive balance test (RBT). This implied a stepwise description of the 1) definitions and exploration throughout the theoretical constructs of balance and adaptability, followed by the 2) development of test items which lead to the creation of the final test protocol.

### The theoretical constructs of balance and adaptability

#### Balance

Despite the widespread use of the term balance, no unanimous consensus exists on its definition. It is often associated or misinterpreted with concepts like stability, equilibrium or postural control [22]. We demarcated balance as being part of human postural control, and defined it as the act of maintaining, achieving or restoring a state of balance during any posture or activity [22]. From this definition balance can be seen as the result of multiple components that interact non-linearly to achieve a mutual goal. Balance and equilibrium are very similar concepts but slightly different from a mechanical point of view. Both refer to an absolute state, whereas stability is a relative quantity. Although not limited to these, the primary components that interact to maintain balance are the visual, vestibular somatosensory and neuromuscular system [22, 23]. Balance can be objectively measured by either reach distance (e.g. star excursion balance test, Y-balance test) or by counting balance errors when performing a balance test (e.g. balance error scoring system). Both reach distance and balance errors have been proven to be reliable and valid outcome measures [19, 24–26]. Current balance tests aim to measure pre-planned motor responses and focus on the neuromuscular system which uses information from the somatosensory and vestibular system (e.g. star excursion balance test, Y-balance test). However, this neglects the importance of balance in its relevant context of sports performance or injury prevention. Within this context, adaptability has been put forward as a concept to understand how balance during dynamic activity is affected by a changing environment.

#### Adaptability

Adaptability was defined as the ability to effectively modify responses under a broad spectrum of conditions [15]. A subjects’ adaptability is characterised by the robustness of the balance system as in his or her ability to maintain postural control in a changing environment whilst executing a motor task [15]. This highlights the importance of evaluating balance coupled to unanticipated stimuli and goal-oriented motor tasks when exploring functional tests within the construct of adaptability. This implied the need to implement a visuomotor task (VMT) involving uncertainty and decision-making to integrate the broad spectrum of possible responses in the RBT. Adaptability in this context requires signal processing and decision-making in the form of cognitive demands, coupled to motor execution, which can be objectified by the visuomotor reaction time [27].

The visuomotor reaction time on a behavioural level considers the time needed from start to end-point of the VMT. In detail, this pathway includes visual perception, visual processing, visuomotor transformation and motor execution time [27]. In cognitive research, pre-motor time includes all but the motor execution time which can be used to gain insights in time needed for signal processing solely [27]. In the context of adaptability, it was more relevant to consider the visuomotor reaction time on the behavioural level as the time needed to select and execute the appropriate motor response in a changing environment. Previous research showed that together with the reaction time and pre-motor time as outcomes for speed of signal processing, accuracy can be used as an outcome measure of adequate signal processing and learning [28]. This allowed for both visuomotor reaction time and accuracy as outcome measures to gain insight in a subject’s adaptability, with visuomotor reaction time scoring the subjects speed to select and execute a motor response and, accuracy scoring the rate to which the motor response was correct.

In conclusion, these insights showed that the concepts of balance and adaptability are compatible and reinforcing in a real-world context. A functional test that provides objective measures within both concepts would be beneficial to aid clinicians to map the rehabilitation progress and substantiate criterion based return to sport decisions.

### Developing test items for the reactive balance test (RBT)

The development of a functional test designed to assess a subject’s ability to maintain balance and adequately react to environmental stimuli should go through different phases in order to provide an added value for clinicians and researchers:

1. The selection of an appropriate balance test
2. Integrate adaptability – designing the VMT
3. Provide an objective measure of balance ability
4. Provide an objective measure of adaptability

#### The selection of an appropriate balance test

The validity and reliability of the star excursion balance test (SEBT) and Y-balance test (YBT) have been thoroughly investigated as a measure of (dynamic) balance both in healthy and musculoskeletal injured populations (e.g. anterior cruciate ligament rupture, chronic ankle instability). Both are used in clinical practice to monitor the rehabilitation progress or to identify high risk athletes via screening [2, 19, 26, 29–33]. Outcome measures that are used to score a person’s balance ability are balance errors and/or reach distance [2, 19, 26, 29–33]. Although multiple balance tests exist, it is important to understand the similarities and differences in test names and standardization of test methods. The SEBT is a balance test encompassing a star based shape with 8 directions. This test has been modified and named the modified-SEBT when only comprising 3 axes of the star based shape. Within this test spectrum, the YBT is a balance test with 3 directions in a Y-shape with the anterior axis pointing straight forward and a posteromedial and posterolateral axis positioned in a 135° angle from the anterior axis. The YBT was the only balance test that was included in the systematic review of Hegedus et al. (2015) with documented excellent criterion validity and good reliability [2, 19]. During the YBT, the participant is instructed to stand on one leg on the central platform and to reach as far as possible along the anterior, posteromedial, and posterolateral axis by pushing a reach indicator (Y Balance Test Kit™, FunctionalMovement.com, Danville, VA).. Moreover, the YBT has been previously used and adapted by Batson in 2010 in order to build a more sport specific balance test for dancers, with continuing research on its applicability by clinicians by Wilson and Batson (2014) [34, 35].

The YBT was selected as the base of our functional test given its predicate as a clinician-friendly performance test, its moderate to good reliability as a balance test [19, 26, 31, 33], previous modifications [7, 34, 35] and the notion that this test allowed for visuomotor reaction time to be measured and balance errors to be counted throughout the test. Furthermore, instructions and recommendations for standardized protocol with the provided rationale for every recommendation for the YBT were adopted from Plisky and colleagues (2009) [19] and were applied to the RBT.

#### Integrate adaptability – designing the VMT

Given the aforementioned definition of adaptability [15], the test design implied the need of using unanticipated stimuli. Previous research has used a VMT and the visuomotor reaction time to gain insights in the neural correlates of visuomotor reaction time in badminton players [27]. Although a VMT was feasible to determine visuomotor reaction time using unanticipated stimuli, it needed to include as much uncertainty as possible to broaden the spectrum of possible conditions. To maximise cognitive load and the need for the subject to select the appropriate motor response, a multi-layered approach was thus warranted to allow uncertainty and adequate cognitive and motor responses at the start, throughout the execution and at the end of the task. Therefore, the VMT was developed with the visual stimulus being random in sequence and interstimulus time (1.5 s, 2 s or 2.5 s) and the result of the VMT being discrete in its pass or fail nature.

The cognitive process was designed using an initial visual stimulus in the colour red, blue or green whereby each of the three axes of the YBT corresponded to a specific colour of the visual stimulus. The colour blue was assigned to the anterior axis, the colour red to the right posterior axis and the colour green to the left posterior axis. Colours were fixed to spatial orientation because this eliminated corresponding cognitive errors. The initial visual stimulus needs to be processed to determine the direction of the correct motor response. LED-lights (FitLight training system^TM^) were used for providing the visual stimuli. The order of the stimuli was randomized as well as the interstimulus time. This avoided the athlete anticipating both the timing of the next visual stimulus and the direction of the targeted motor response. To obtain an objective measure of visuomotor reaction time data corresponding to each axis, the number of reaches per axis needed to be maximized. This would have prejudiced the ecological validity of the test, so the choice was made to set the number of stimuli per axis to as much as possible without the duration of the test exceeding two minutes. The cognitive decision-making process could be intensified by reducing interstimulus time or using more than 36 stimuli to put a greater strain on focus and attention, as well as on balance errors since the participant would get less time to correct perturbances of balance. Reducing the interstimulus time to less than 1.5 s, however, would not allow the subject to return to the starting position of the test after extinguishing the preceding sequence. To ensure the ecological validity of the RBT, the decision was made to use the minimal number of stimuli needed and to focus the load of the cognitive process on randomization of both the order of the stimuli as the interstimulus time, with the interstimulus time being as low as possible ranging between 1.5 and 2.5 s.

In conclusion, a VMT task using 12 randomised stimuli along each axis of the YBT was developed to reach a test duration of 90 to 120 s. Stimuli were randomised in sequence and interstimulus time to maximise uncertainty and avoid anticipation. The ability of the subject to successfully extinguish the LED-light was scored by success or fail.

#### Provide an objective measure of balance ability

Reach distance and balance errors are valid and reliable outcome measures to score balance ability [2, 19, 26, 29–33]. Since the YBT was chosen as the base of the RBT, the challenge was to select the optimal balance between reach distance and balance errors throughout the RBT. The RBT uses a fixed reach distance along each axis. If the reach distance was set to maximal, subjects would fail the VMT in its success or fail outcome. If reach distance was set too low, no balance challenge would be provided. To provide an objective measure of balance ability, the balance error scoring system (BESS) can be used throughout the test [25]. A balance error was defined as a deviation from the proper stance, which can result in a minor balance error or a major balance error. A minor balance error was a deviation from the proper stance that did not obstruct the subject to continue the test sequence. A major balance error was a balance error that was important enough to cause a delay in the visuomotor reaction time or cause the subject to not be ready for the next stimulus (see Table 1). To maintain validity as a balance test, the RBT needed to stress the complex interactions between the visual, vestibular, somatosensory, and neuromuscular system that result in balance.

Therefore, the choice was made to set the reach distance during the RBT to minimally 80% of the maximal reach distance in order to not cause unnecessary major balance errors and to enable the subject to timely continue the test sequence.

#### Provide an objective measure of adaptability

Given the aforementioned definition of adaptability [15], the test design implied that visuomotor reaction time and accuracy were essential outcome measures to score the efficiency and efficacy of the RBT [36].

##### Visuomotor reaction time

Visuomotor reaction time is a measure of processing speed and reflects response efficiency in information processing tasks necessary in fast paced sports [36]. In response to a visual stimulus, visuomotor reaction time is used at a behavioural level to describe the time needed from start to end-point of the VMT [27]. The inherent relation of adaptability, balance and visuomotor reaction time allow an athlete to execute the desired motor response in a changing environment. To allow for an objective measure of visuomotor reaction time, the RBT uses photosensor technology as incorporated in the Fitlight training system^TM^. Visuomotor reaction time measured with the Fitlight trainer system^TM^ has proven to be a reliable method when using cognitive tasks and coupled motor responses, and is able to discriminate between elite and non-elite players [37].

If the LED-lights were placed at maximal reach distances, subjects could either be unable to reach the set distance resulting in a loss of accuracy or lose balance while trying. This total loss of balance would then impede the subject in the test sequence to prepare for the next stimulus. Both scenarios were undesirable when both visuomotor reaction time and balance errors were selected as outcome measure. Thus, to provide an objective measure of the average visuomotor reaction time during the RBT, the choice was made to set the reach distance to 80% of the maximal reach distance of the corresponding YBT axis. We hypothesized that this would adequately stress balance but would avoid unnecessary errors in accuracy.

##### Accuracy

Accuracy can be used as an outcome measure of adequate signal processing and learning [28] and relates to both the execution of the motor task as the associated cognitive process. By combining the YBT with a VMT, accuracy of the motor task can be used as an outcome measure because of the pass or fail nature of the RBT. Furthermore, the accuracy of the cognitive process can be described using decision errors during the RBT. The challenge within the design of the RBT, was setting the reach distance to such distance that the balance system was stressed, but the targeted motor response could still be swiftly executed. Accuracy is a measure that describes the success rate of the subject in extinguishing the correct LED-diode within the given timeframe. To be accurate in clinical practice, the subjects needs to observe and interpret the visual stimulus and execute the correct motor response. The sole measure of accuracy would thus not have incorporated time pressure relating to the targeted motor response. Given the prior description of the instruction to execute the targeted motor response as fast as possible, the timeframe needed to allow enough time for the motor execution. The maximal timeframe set for the subject to extinguish the LED-diode was 2 s, because the most plausible explanation of a subject not being able to extinguish the LED-diode was because of a major loss of balance. This timeframe of 4.5 s (maximal stimulus time + maximal interstimulus time) allowed the subject to timely reposition for the continuing test sequence after losing complete balance.

An added outcome measure of accuracy can be coupled to the cognitive process and accompanying decision errors. A decision error was defined as the participant initiating movement in the wrong direction as indicated by the visual stimulus and thus recognized by the faulty start of an incorrect motor response. Using video-analysis, the direction in which the motor response was started could be observed and compared to the correct motor response direction as set by the visual stimulus. Discrepancies could be counted as a decision error in the cognitive interpretation of the visual stimulus, whereby the motor response could be determined as being inaccurate and the corresponding visuomotor reaction time could be extracted from the visuomotor reaction time analysis.

Therefore, the interstimulus time was set to vary between 1.5, 2 and 2.5 s to allow swift test progress and still integrating time pressure. Accuracy could be scored counting the fail results of the VMT. The 80% reach distance allowed to stress the balance system, without deviations along the YBT axis causing loss of accuracy. The RBT allowed for the added possibility to use video-analysis to score decision errors, when the subject still successfully extinguishes the LED-diode after initiating a faulty motor response.

Therefore, the accuracy of the RBT was scored by counting the missed stimuli and multiple attempts, as well as by documenting decision errors throughout the RBT.

## Results

### Test protocol: RBT

**Fig. 1:**
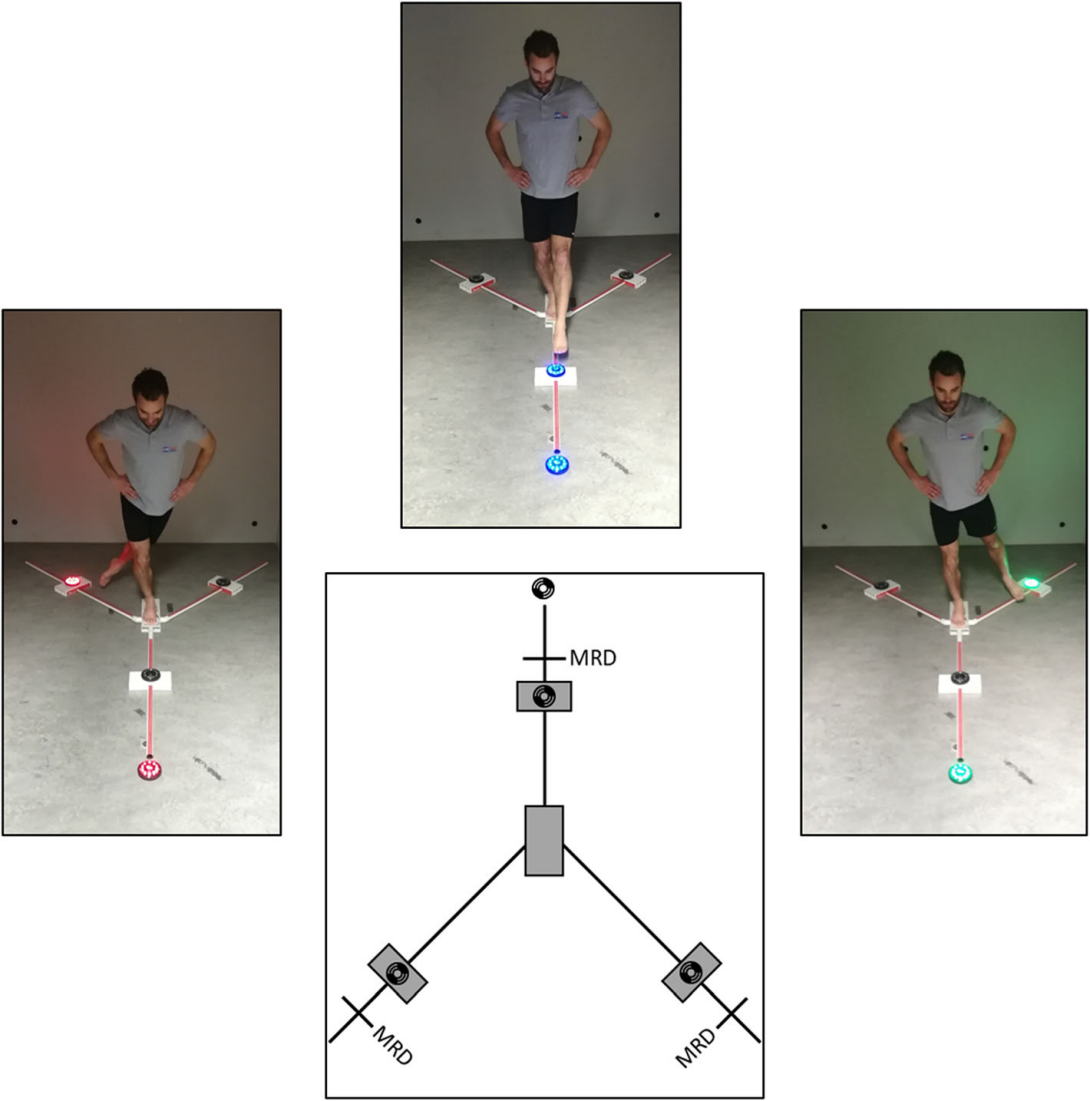
Reactive balance test. MRD = Maximal Reach Distance;  = Fit-light trainer^TM^ LED-lights. The LED-lights are placed on the axes of the Y-balance kit at 80% of the MRD. Also, each LED-light on every axis has a designated colour (e.g. blue = anterior axis). The LED-light in front of the Y-balance kit randomly shows one of the corresponding colours and indicates in which direction the participant has to reach as fast as possible and without losing balance

The test incorporated the Y Balance Test KitTM in combination with Fitlight-training system^TM^ (see Fig. 1). Instructions and recommendations for standardized protocol were adopted from Plisky and colleagues (2009) and were also applied to the RBT [19]. One LED-light was placed in front of the YBT and three LED-lights were placed on the Y Balance Test KitTM at 80% of each participant’s maximal reach distance. Participants had to take on the YBT standardized starting position (see above). The LED-light in front emitted for 0.2 s one of three selected colours (red, blue, or green), and was always followed instantaneously by a colour-matched LED-light attached to the Y Balance Test KitTM for 2 s. Subjects were instructed to extinguish the corresponding emitting LED-light attached to the YBT axis as fast as possible by passing over the LED-light with one’s foot within a range of 5 cm without losing balance. The 36 visual stimuli of the VMT occurred in a predetermined, but randomised sequence (http://www.randomization.com). The inter-stimulus time varied between 1.5, 2, or 2.5 s to eliminate anticipatory timing effects, provide enough challenge for the test subject and give enough time to recover the standard position when a balance or decision error was made. Furthermore, the starting point of the colour sequence was randomised for every performed RBT, so participants could not memorize the colour sequence, nor the inter-stimulus times when performing the test multiple times (e.g. alternating between left and right stance leg).

**Table 1:** Reactive balance test outcome measures

Visuomotor Reaction Time = averaged total visuomotor reaction time	
Accuracy = (Total number of stimuli – (missed stimuli + multiple attempts needed + decision errors))/100	
- *Missed stimulus* = failed to extinguish LED-light - *Multiple attempts* = reaching from standardized position, but failed to extinguish the LED-light from the first time; second or third attempt needed - *Decision error* = initiating movement in wrong direction	
Balance error = number of balance errors [25] - *Minor balance error* = looking for balance but able to start from standard position at stimulus onset or looking for balance during reach - *Major balance error* = not starting from standard position at stimulus onset or during stimulus presentation caused by hand or foot on floor; stepping off the YBT - *Predefined balance errors =* moving hands off the hips; step, stumble or fall; Abduction or flexion of the hip while looking for balance; lifting the forefoot or heel off the testing surface; placing the free foot on the floor; remaining out of the proper testing position for greater than 2 s	

In summary, the outcome measures of the RBT are visuomotor reaction time, accuracy and balance errors. A detailed description of all outcome measures of the RBT is listed in Table 1 and recommendations for a reactive balance test protocol can be found in Table 2.

**Table 2:** Recommendations for reactive balance test protocol

Recommendation	Rationale
Randomize order of stimuli	Avoids stimulus anticipation for direction by subject
12 stimuli per axis	As much as possible to improve reliability of visuomotor reaction time without exceeding a two minute test duration
Randomized interstimulus time	Avoid stimulus anticipation for timing by subject
80% reach distance	Balance perturbation, without the intend to impair accuracy
80% reach distance	Balance perturbation, without the intend to cause balance error that discontinues test sequence

## Discussion

This test protocol described a reactive balance test that added environmental perception, decision-making and variable motor responses as additional dynamic components to the construct of balance. Given the importance of adaptability in sports performance, this functional test can be of added value in the functional test repertoire to objectify the progress throughout rehabilitation or support return to sport decisions. The development of this test was a first step in incorporating the insights of adaptability into functional testing, but is still a long way removed from a real sports environment. Yet, this test could easily be used in clinical practice during the rehabilitation process as one of the first objective indicators of an athlete’s ability to maintain balance in a changing environment. The RBT involved different levels of uncertainty and tests the athlete’s capacity regarding the combined components of decision-making, balance and visuomotor reaction time. The multi-layered approach of this test allowed for multiple outcome measures to be described, based on its construct validity. Visuomotor reaction time and accuracy were frequently used outcome measures in research towards neurocognitive functioning, often related to a non-specific task [38–43]. In clinical practice, a delayed neurocognitive reaction time indicates an increased injury risk for lower extremity sprains and strains [18]. These injury populations are also known to have a reduced balance ability, which is also a strong predictor of first-time sprains and recurring sprains [8–11, 44–46] and warranted a concurrent approach of visuomotor reaction time and balance outcomes to monitor the rehabilitation process. Balance errors are a reliable outcome to score balance [25], but are susceptible to interpretation. Predefining balance errors is a crucial step in both research as well as clinical practice. Our test protocol incorporated previous insights both in balance test protocol [19] as in predefined balance errors [25] with the added label of minor versus major balance errors. This label allowed to reduce errors in human judgement and improved reliability of measures as documented in scapular dyskinesis research [47]. Clear definitions for major and minor balance errors were also provided for the RBT. Estimated total cost of the equipment used in the RBT is predominantly determined by the cost of the system used to run the VMT and evaluate VMRT. These systems however are also used for training and rehabilitation purposes, with prices ranging from €3000–6000 for the system we used. Other equipment used in the RBT includes a Y-balance test kit and video camera. If the Y-balance test kit is not available, paint or tape suffice to create the Y-shaped test design and measure reach distance along the axes.

### Limitations

This paper described the theoretical construct and validity of a RBT. It was only a first step to introduce adaptability and a corresponding clinician-friendly functional test into the functional testing repertoire of clinicians and researchers and needs further research. To support the use of this functional test, validity and reliability of test outcomes need to be researched in the future. Consequently, the added value of this test within the emerging research can grow with possible indications throughout the clinical decision-making and the sports injury prevention research domain. It is important to note that significant learning can be expected in balance tests, including the YBT and RBT. Given the matter that reliability and measurement error of the YBT is still under debate, no recommendation can be given towards the number of practice trials for the RBT. In addition, reliability, standard error of measurement and number of practice trials needed to eliminate learning effects in both YBT and RBT need to be studied in future research in order give proper recommendations. Future research should also investigate both the test properties of the YBT when drawn or taped as well as the test properties when using the YBT test kit, since an important difference in performance can arise due to the different materials used to build the test. Another consideration that we have to keep in mind is possible colour-blindness of the patient or participant. Therefore, the clinician or researcher administering such a test should always check for colour vision deficiency prior to administering such a test and change the colours of the LED-lights accordingly.

## Conclusion

This paper described a test protocol and construct validation for a reactive balance test within the theoretical constructs of balance and adaptability. This clinician-friendly test can be applied in clinical practice and in future research. The RBT used the YBT protocol and implemented adaptability using a VMT. Outcome measure of the test are balance errors, visuomotor reaction time and accuracy. This was a first step within the functional testing repertoire to score an athlete’s adaptability during a balance test.

## Abbreviations

BESS: Balance error scoring system
MRD: Maximal Reach Distance
RBT: Reactive balance test
SEBT: Star excursion balance test
VMT: Visuomotor task
YBT: Y-balance test
